# Supplementary material for: Aromatase deficiency in hematopoietic cells improves glucose tolerance in male mice through skeletal muscle-specific effects
Source: PLoS One. 2020 Jan 23;15(1):e0227830. doi: 10.1371/journal.pone.0227830 (PMC6977739; doi:10.1371/journal.pone.0227830)
Supplement: Attachment — Submitted filename: Response to Reviewers.docx [file pone.0227830.s002.docx]

*We thank the reviewer for additional suggestions for our paper. Please find below our itemized responses to the reviewer’s comments.*

**Comments to the Author**

Reviewer #1: The authors have attempted to address each of the editor’s and reviewer’s concerns and is greatly improved as a result, particularly with regards to the over-interpretation of non-significant data in the first version of the manuscript.

Minor comments;

It is worth noting in the text, that the skeletal muscle gene expression data was expressed relative to beta-actin as it was demonstrated to be the most stably expressed housekeeping gene of those analysed.

We agree that this additional information is important to include and have added the following sentence to our Methods section (lines 209-211):

*Expression of 3 candidate housekeeping genes was measured for each tissue, and the genes exhibiting the most stable tissue-specific expression were utilized for data normalization.*

It is also worth acknowledging in the text that the gene expression data in skeletal muscle could be further supported by protein analyses, however, this was not possible using the current cohort of samples due to the method in which the tissues were processed.

We now include this limitation of our study in our discussion, as follows (lines 480-482):

*Protein analyses also will be important to support the gene expression findings in our study; protein analyses were attempted for this cohort of animals but were not successful due to the methods employed for tissue processing at animal sacrifice.*

Line 342 – The use of the word “trend” should be avoided to describe data with a P value of P=0.09. The description of these data could be re-worded to state that the mean value of ERalpha mRNA was higher in the WT(ArKO) mice than the WT mice however this did not reach statistical significance (P=0.09).

We appreciate this comment, and the wording of this sentence has been amended as suggested (lines 336-338):

*In WT(ArKO) mice, mean expression of ERα (Esr1) was higher relative to WT(WT) mice, although this difference did not achieve statistical significance (p=0.09).*

Lines 367-368 and Lines 439-441 – Similarly, this sentence should be reworded to state that the mean 17B estradiol levels were lower in WT(ArKO) mice, but this did not reach statistical significance P=0.08. The word “quite” in this sentence implies a P value close to 0.05 (ie. such as 0.06), so should be removed.

The word ‘quite’ has been removed in both of these places, as advised (lines 362 and 434).
